# The Use of Radiation Therapy in Well-Differentiated Soft Tissue Sarcoma of the Extremities: An NCDB Review

**DOI:** 10.1155/2015/186581

**Published:** 2015-05-07

**Authors:** Alexander L. Lazarides, William C. Eward, Paul J. Speicher, Chun-Han Hou, Daniel P. Nussbaum, Cindy Green, Dan G. Blazer, David G. Kirsch, Brian E. Brigman

**Affiliations:** ^1^School of Medicine, Duke University, Durham, NC 27710, USA; ^2^Department of Surgery, Division of Orthopedic Surgery, Duke University Medical Center, Durham, NC 27710, USA; ^3^Department of Surgery, Duke University Medical Center, Durham, NC 27710, USA; ^4^Department of Orthopedic Surgery, National Taiwan University Hospital, Taipei 10617, Taiwan; ^5^Department of Radiation Oncology, Duke University Medical Center, Durham, NC 27710, USA

## Abstract

*Objective*. This study investigated patterns of utilization of radiation therapy (RT) and correlated this with overall survival by assessing patients with well-differentiated soft tissue sarcoma of the extremity (STS-E) in the National Cancer Database (NCDB). * Methods*. All patients diagnosed with well-differentiated STS-E between 1998 and 2006 were identified in the NCDB. Patients were stratified by use of surgery alone versus use of adjuvant RT after surgery and analyzed using multivariate analysis, Kaplan-Meier analysis, and propensity matching. *Results*. 2113 patients with well-differentiated STS-E were identified in the NCDB for inclusion with a mean follow-up time of 74 months. 69% of patients were treated with surgery alone, while 26% were treated with surgery followed by adjuvant RT. Patients undergoing amputation were less likely to receive adjuvant RT. There was no difference in overall survival between patients with well-differentiated STS treated with surgery alone and those patients who received adjuvant RT. *Conclusions*. In the United States, adjuvant RT is being utilized in a quarter of patients being treated for well-differentiated STS-E. While the use of adjuvant RT may be viewed as a means to facilitate limb salvage, this large national database review confirms no survival benefit, regardless of tumor size or margin status.

## 1. Introduction

Soft tissue sarcomas (STS) are a heterogeneous group of rare tumors that share a mesenchymal origin. Despite a low incidence of less than 1% of new cases per year [1, 2], sarcoma is disproportionately lethal, which is mostly attributable to the development of pulmonary metastasis [3]. Established prognostic factors for poor outcomes in patients with STS of the extremities (STS-E) include grade, size, depth, and patient age [4, 5]. Of these, tumor grade is probably the most sensitive indicator of a tumor's biological behavior and the strongest predictor of metastasis and death from disease [6, 7]. Well-differentiated tumors have dramatically different biological behavior from their intermediate- and high-grade counterparts. Patients with well-differentiated STS-E have a much lower likelihood of developing metastasis and dying from disease. For such patients, the role of adjuvant radiation therapy (RT) has been to limit local recurrence rather than have an impact upon survival [8, 9].

When adjuvant RT should be utilized in the treatment of well-differentiated STS-E, or if it should be utilized at all, it remains a matter of controversy. The goals of treatment are achieving local control, preserving function of the involved limb, and reducing the risk of death from disease. Radiation therapy is regarded as unlikely to reduce the already low risk for death from disease and it has the potential to worsen function of the involved limb. For these reasons, some physicians believe that the risks of RT outweigh its potential benefits with regard to well-differentiated STS-E [10]. In a recent review of the SEER database, Koshy et al. reported that adjuvant radiation utilization was associated with a survival benefit for high grade, but not low grade STS [11].

At present, the National Comprehensive Cancer Network NCCN guidelines state that, regardless of depth, low grade tumors less than 5 cm should be treated with surgery alone; consideration may be given for RT if margins are inadequate, which is defined by the NCCN as a goal of margins >1 cm [12]. Low grade tumors greater than 5 cm should be treated with surgery and RT for all patients whose margins are inadequate. Given that an exact amount of margin that is sufficient has never been determined, it is probably impractical to make blanket recommendations for RT utilization, especially for low-grade tumors where survival is not likely to be affected. Using the large patient cohort with well-differentiated STS captured by the National Cancer Database (NCDB), the goals of our project were to investigate patterns of utilization of RT with regard to several known prognostic factors and to correlate the use of RT on survival in this subset of patients.

## 2. Methods

The Duke University Institutional Review Board approved this retrospective analysis of the American College of Surgeons/American Cancer Society National Cancer Data Base (NCDB). More than 1500 Commission on Cancer- (CoC-) accredited facilities contributed to this registry, accounting for a large majority of new cases of cancer every year in the USA. To identify patients who underwent resection of an extremity soft tissue sarcoma, the NCDB Participant User File for 1998 through 2011 was utilized first queried for all patients treated at a NCDB participating institution for tumors in the arms or legs with International Classification of Diseases for Oncology, 3rd Edition (ICD-O-3) topography codes C47.1, C47.2, C49.1, and C49.2. Relevant histologic subtypes were selected based on a series of ICD-O-3 histology codes, all of which represented soft-tissue sarcomas. Other inclusion criteria included malignant behavior, primary cancer diagnosis, no distant metastasis, and known status for preoperative radiation therapy.

National trends in the rate of adjuvant RT, defined by the NCDB as “radiation therapy given after surgery to the primary site,” were examined with the Cochran-Armitage trend test in patients with well-differentiated soft tissue sarcomas of the extremities. The use of adjuvant RT was then used to classify subjects into two groups. Baseline characteristics and outcomes between groups were compared using Pearson's chi-square test for categorical variables and analysis of variance (ANOVA) for continuous variables. Multivariable logistic regression was used to predict factors that were associated with the administration of adjuvant RT. Patients who received neoadjuvant RT, defined by the NCDB as “radiation therapy given before surgery to the primary site,” were excluded to avoid potential biases in a comparison of sarcomas graded from a biopsy prior to preoperative radiation therapy with sarcomas graded from the entire resected tumor. Furthermore, grading sarcomas after preoperative radiation therapy and resection could be altered by radiation effects. To control for confounding in the use of adjuvant RT, we used propensity matching, which is defined as “conditional probability of assignment to a particular treatment given a vector of observed covariates” [13]. Using this system, we developed propensity scores, which were defined as the a priori conditional probability of being treated with RT prior to surgery. Patients were then matched on these propensity scores, using a 1 : 1 nearest neighbor algorithm, which included the following variables: age, sex, race, Charlson/Deyo comorbidity score, patient census tract education and income levels, tumor size, histologic subtype, histologic grade, tumor location (upper versus lower extremity), treatment facility type (academic or community hospital), and extent of resection. Adjusted medians and proportions between the propensity-matched groups were then compared. With propensity-matched analyses, we hoped to employ a method that corrects, at least partially, for confounding factors.

To ensure the accuracy of survival data, the NCDB only provides vital status for patients five years following the date of surgery. Therefore, survival from the time of diagnosis was assessed for all patients who underwent resection prior to 2007. Prior to analysis, subjects who underwent resection from 1998 to 2006 were rematched using the aforementioned variables. Then, long-term survival among groups was evaluated using the Kaplan-Meier method with comparisons based on the log-rank test.

A more specific analysis was carried out for patients with margin negative tumors and margin positive tumors. Within these groups, an analysis was carried out for size smaller than 5 cm and size greater than 5 cm. The above analyses were again repeated with patients with well-differentiated liposarcoma of the extremities only. It is not possible to determine rates of local recurrence for patients with STS-E enrolled in this database.

Results are reported as median (IQR), proportions (%), and odds ratios (OR, 95% CI) as applicable. *p* values < 0.05 indicate statistical significance, and we controlled for type I error at the level of the comparison. All statistical analyses were performed using R (the R Foundation for Statistical Computing, version 3.0.2, Vienna, Austria).

## 3. Results

### 3.1. Demographics and Patient Characteristics

A total of 2340 patients were identified in the NCDB who had undergone resection of an STS-E with a well-differentiated histologic grade. Five hundred seventy-three patients (27%) received adjuvant RT; 1540 patients (73%) were treated with surgery alone. A small number of patients (*n* = 98) who received neoadjuvant RT or combined neoadjuvant and adjuvant (*n* = 12) were excluded due to the potential challenge in grading the sarcoma following definitive resection because of radiation effect. Furthermore, 98 patients had missing RT treatment data and were therefore excluded from the study, leaving a remaining 2113 patients to be evaluated.

Baseline characteristics are shown in Table 1. There were no statistically significant differences between groups with regard to age, sex, distance to cancer center, preoperative comorbidities, income, or education. Additionally, there were no statistically significant differences in Charlson Comorbidity Scores between the two groups.

### 3.2. Tumor Characteristics and Treatment Choices

With regard to tumor characteristics, patients who received adjuvant RT were more likely to have tumors larger than 5 cm (*p* < 0.001, Table 1). When categorized by histologic subtype, adjuvant RT was more commonly utilized in patients with liposarcoma (68.2% receiving adjuvant RT versus 64.8% receiving surgery alone), malignant fibrous histiocytoma (5.6% versus 4.5%), MPNST (3.4% versus 2.8%), and myxosarcoma (1.7% versus 0.6%) when compared to other histologic subtypes. Patients who underwent adjuvant RT were also more likely to have received adjuvant chemotherapy than those who did not receive adjuvant RT (6% versus 0.4%, *p* = 0.005). They were also more likely to undergo radical resections (65.7% versus 62.4%, *p* = 0.002) than local excisions. Of the 69 patients treated with an amputation, 64 of those were treated with surgery alone. Only 5 patients (0.8% of the RT group) were managed with a combination of amputation and adjuvant RT. Patients with microscopically or macroscopically positive margins were more likely to receive adjuvant RT (26.7% versus 19.5%, *p* = 0.001) (Table 1). A multivariable logistic regression analysis found no independent predictors of adjuvant RT use (Table 1). There were no differences in surgery type, surgical margins, 30-day mortality, readmission rates, or hospital length of stay.

### 3.3. Survival Outcomes

**Figure 1:**
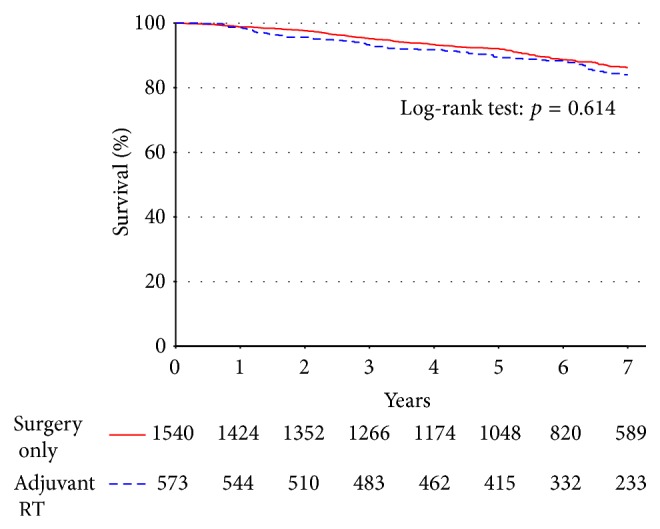
There is no difference in unadjusted survival for patients with well-differentiated tumors when stratified by adjuvant RT versus surgery alone.

Five-year survival from the time of diagnosis for all patients (*n* = 2113; Figure 1(a)) was not statistically different between patients treated with surgery alone and patients receiving adjuvant RT (89.5% versus 92.1%, *p* = 0.614). These patients were rematched on the propensity to receive adjuvant RT, adjusting for confounding variables (Table 3). Again, the analysis showed no statistically significant differences in survival (89.5% versus 92.2%, *p* = 0.984).

**Figure 2:**
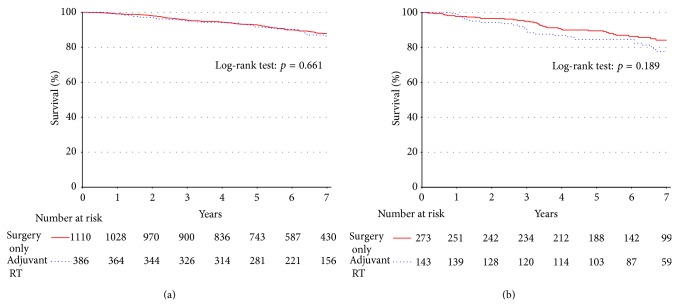
There is no difference in unadjusted survival for (a) patients with well-differentiated tumors when stratified by adjuvant RT versus surgery on the basis of negative margins. There was also no difference when stratified on the basis of positive margins (b).

For patients who had a negative surgical margin (*n* = 1496; Figure 2(a)), there was no statistically significant difference in 5-year survival rates based on the addition of adjuvant RT (91.6% versus 94.5%, *p* = 0.887). These patients were rematched on the propensity to receive adjuvant RT, adjusting for confounding variables (Table 3). This showed no statistically significant survival difference for patients treated with adjuvant RT (91.6% versus 94.5%, *p* = 0.85). For patients who had a positive surgical margin (*n* = 416; Figure 2(b)), there were also no statistically significant differences in survival rates for patients treated with adjuvant RT (84.4% versus 89.4%, *p* = 0.189). These patients were rematched on the propensity to receive adjuvant RT, adjusting for confounding variables (Table 3). No statistically significant survival difference was shown for patients treated with adjuvant RT (84.4% versus 92.4%, *p* = 0.096).

**Figure 3:**
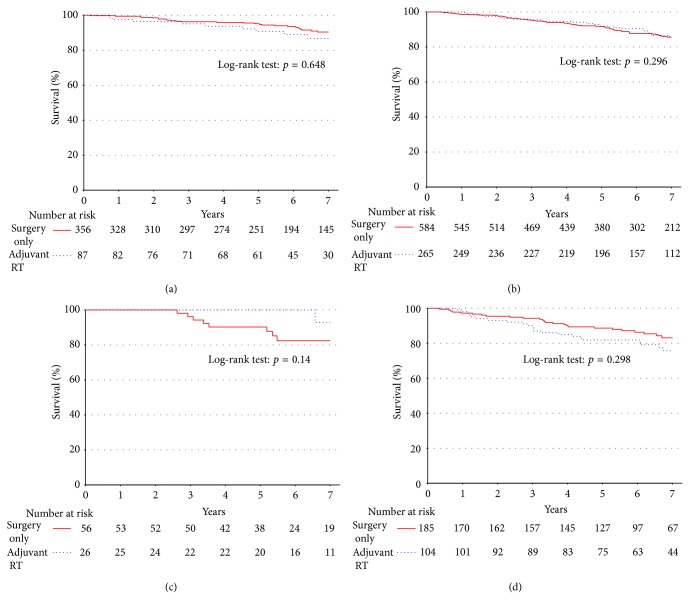
There is no difference in unadjusted survival for patients with well-differentiated tumors when stratified by adjuvant RT versus surgery on the basis of size <5 cm (a) and >5 cm (b) for patients with negative margin status. There was also no difference when stratified on the basis of size in patients with positive margin status ((c) and (d), resp.).

For patients with large (greater than 5 cm) tumors, use of adjuvant RT had no influence on survival. Survival was plotted among all patients who had margin-negative, well-differentiated tumors less than 5 cm (*n* = 464; Figure 3(a)) and greater than 5 cm (*n* = 862; Figure 3(b)). This revealed no statistically significant difference in survival rates for patients receiving the adjuvant RT (90.7% versus 95.1%, *p* = 0.648 and 91.9% versus 91.8%, *p* = 0.296), respectively, for these groups. A similar analysis was carried out for patients who underwent surgery who had margin-positive well-differentiated tumors less than 5 cm (*n* = 84; Figure 3(c)) and greater than 5 cm (*n* = 291; Figure 3(d)). There were no statistically significant differences in survival rates for patients receiving adjuvant RT compared to those who did not (100% versus 90.2%, *p* = 0.14 and 81.8% versus 88.6%, *p* = 0.298) in each of these two groups.

### 3.4. Well-Differentiated Liposarcoma

Because the well-differentiated liposarcoma is its own unique and common subtype [14], we repeated our analysis to evaluate the large group of patients with this tumor. Out of the initial 2157 patient cohort, a total of 1418 patients were identified who had undergone resection of a well-differentiated liposarcoma of an extremity. Subjects were again grouped by surgery with adjuvant RT (404 patients, 28%) versus surgery alone (1014 patients, 72%). Baseline characteristics are shown in Table 2. Patients who underwent adjuvant RT were more likely to be older (57 versus 54 years old, *p* = 0.006). There were no statistically significant differences in sex, distance to cancer center, tumor characteristics, preoperative comorbidities, income, education, or Charlson Comorbidity Scores. After surgery, there were no statistically significant differences between groups with regard to margin status, 30-day hospital readmission or hospital LOS (Table 2).

**Figure 4:**
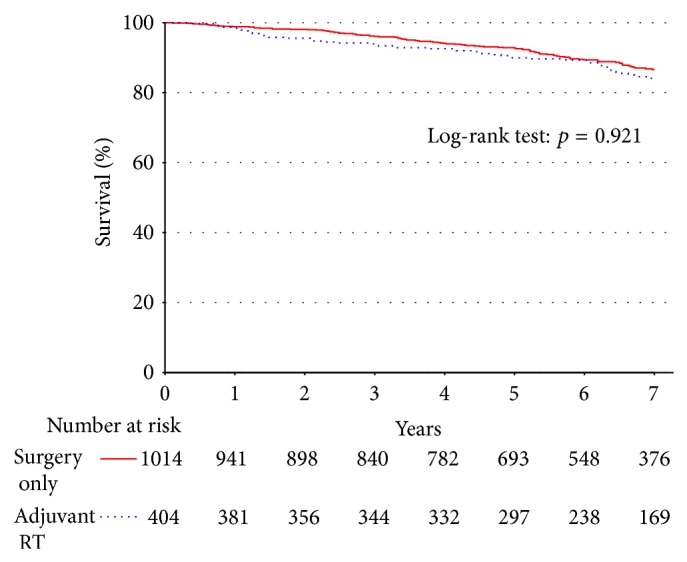
There is no difference in unadjusted survival for patients with well-differentiated liposarcoma when stratified by adjuvant RT versus surgery alone.

**Figure 5:**
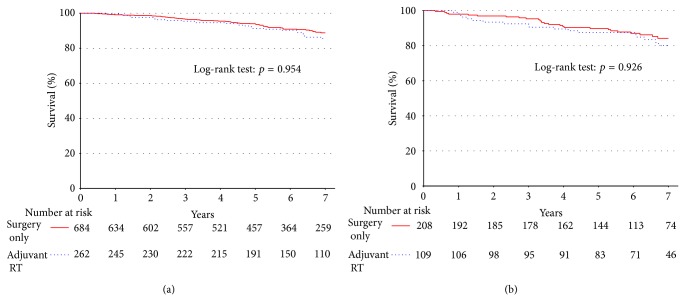
There is no difference in unadjusted survival for (a) patients with well-differentiated liposarcoma when stratified by adjuvant RT versus surgery on the basis of negative margins. There was also no difference when stratified on the basis of positive margins (b).

Survival from the time of diagnosis was evaluated for all patients who underwent resection of a well-differentiated liposarcoma. First, survival was plotted among all patients who underwent surgery during this period (*n* = 1418; Figure 4), which revealed no statistically significant difference in survival rates for patients receiving the addition adjuvant RT (*p* = 0.921). These patients were subsequently rematched on the propensity to receive adjuvant RT, adjusting for confounding variables (Table 3). Again, the analysis showed no statistically significant survival difference for patients treated with adjuvant RT (*p* = 0.84). Survival was plotted among all patients with well-differentiated liposarcoma by margin status, with and without radiation therapy. Both with and without propensity matching to correct for confounding variables, no difference in survival was identified between these groups (Figures 5(a)-5(b), Table 3).

**Figure 6:**
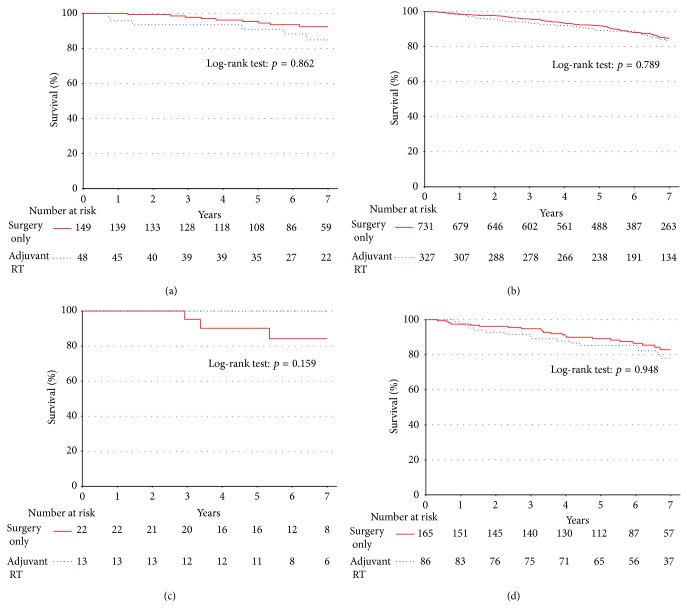
There is no difference in unadjusted survival for patients with well-differentiated liposarcoma tumors when stratified by adjuvant RT versus surgery on the basis of size <5 cm (a) and >5 cm (b) for patients with negative margin status. There was also no difference when stratified on the basis of size in patients with positive margin status ((c) and (d), resp.).

Our final analysis for this group of patients with well-differentiated liposarcoma was aimed at determining whether RT influenced survival when patient data was analyzed on the basis of the size of all well-differentiated tumors. Survival was plotted among all patients who underwent surgery during this period who had margin-negative well-differentiated tumors less than 5 cm (*n* = 148; Figure 6(a)) and greater than 5 cm (*n* = 697; Figure 6(b)). These analyses revealed no statistically significant differences in the survival rates for patients receiving the adjuvant RT for either of these groups (less than 5 cm: 90.9% versus 95.4%, *p* = 0.862 and greater than 5 cm: 89.2% versus 92%, *p* = 0.789). A similar analysis was carried out for patients who underwent surgery who had margin-positive well-differentiated tumors less than 5 cm (*n* = 35; Figure 6(c)) and greater than 5 cm (*n* = 251; Figure 6(d)). Again no statistically significant differences in 5-year survival rates were observed for patients receiving the adjuvant RT in either group (less than 5 cm: 100% versus 90.2%, *p* = 0.159 and greater than 5 cm: 85.4% versus 89.2%, *p* = 0.948).

## 4. Discussion

Soft tissue sarcomas of the extremities (STS-E) represent a heterogeneous group of tumors with a wide variation in biologic behavior. Much of the data that influences treatment decisions is limited by small numbers and by a failure to investigate tumors of a specific grade. Grade is arguably the single most important independent predictor of behavior and well-differentiated STS-E have a limited capacity for metastasis and causing death from disease [15]. With access to the NCDB, this study represents the largest patient cohort to date focusing specifically on patterns of radiation therapy usage and outcomes of patients with well-differentiated STS-E. The primary objective of this retrospective cohort study was to examine the usage patterns of adjuvant radiation therapy in well-differentiated STS-E patients.

The goal of surgery for soft tissue sarcomas of the extremities is en bloc resection of the tumor with a negative margin [16, 17]. However, with surgery alone the rate of local recurrence approaches 40 percent for all sarcomas [18, 19]. The addition of radiation therapy improves the rate of local control to over 90% [20]. While the impact of local recurrence on metastasis and survival remains a subject of debate, local control is an important aspect of sarcoma management, as local recurrences can have major morbidity that may compromise limb function as a consequence of tumor progression or from the need for subsequent surgery and/or adjuvant therapies [21]. One limitation of this study is the ability to determine rates of local recurrence from the NCDB.

A large study conducted of 8249 patients with soft tissue sarcoma of any anatomic location from the Florida Cancer Data System confirmed that low-grade tumors demonstrated a significant survival advantage compared to other sarcomas [22]; they also found that there was no survival benefit with the addition of radiation therapy, though they did not specifically examine by location. A study by Pisters et al. included a cohort of 46 patients with well-differentiated soft tissue sarcomas receiving brachytherapy [9]. Their findings indicated that well-differentiated soft tissue sarcomas exhibited no improvement in local tumor control with the addition of brachytherapy. In contrast, a study by Yang et al. (*n* = 55) found that external beam RT improved the rates of local control in well-differentiated STS of the extremities [8]. Regardless of the impact on local control, neither study showed a survival benefit with the addition of radiation therapy following surgery. Additionally, the studies that demonstrate LR to be an independent risk factor for metastasis and death have not looked specifically at well-differentiated tumors. Some authors have questioned whether or not well-differentiated tumors are even likely to respond to RT, given their latent biology.

With these points in mind, one might wonder why adjuvant RT would be considered at all for patients with well-differentiated tumors. At our institution, adjuvant RT is reserved for those patients with well-differentiated STS-E in whom the morbidity of a subsequent resection would be unacceptable. Therefore, we were surprised to find that 27% of all patients with well-differentiated STS-E in the NCDB were treated with adjuvant radiation therapy. One might expect that this would result from patients with challenging or high-risk disease being disproportionately treated with adjuvant RT. Yet this was not the case. Patients with positive margins received RT 36% of the time and patients with tumors >5 cm received RT 32% of the time. In a separate analysis of the NCDB focusing exclusively on patients with high grade STS-E, we found that RT was utilized only 62% of the time (Hou et al., in press). These data suggest that adjuvant RT is underutilized in patients with high grade STS-E while being overutilized in patients with low grade STS-E. We did find that the use of adjuvant RT for well-differentiated sarcomas was associated with a significantly lower rate of limb amputation as the index procedure (0.8% in the RT group versus, 4.1% in the surgery alone group, *p* < 0.001). This pattern may suggest that surgeons are considering adjuvant RT as a means of limiting the need for amputation. Alternatively, it may simply reflect the fact that patients requiring a limb amputation have little need for adjuvant RT.

We were also intrigued to find that patients who received adjuvant RT were also more likely to have received adjuvant chemotherapy than those who did not receive adjuvant RT. The role of chemotherapy in treating soft tissue sarcoma is controversial. While some data supports a survival benefit for utilizing chemotherapy to treat large, deep, high grade STS [23], there is no evidence supporting the use of chemotherapy to treat low grade STS [24]. In general, the benefit of systemic therapy for soft tissue sarcoma is improved survival, rather than improved local control. A large analysis from the Sarcoma Meta-Analysis Collaboration found that the use of doxorubicin and ifosfamide improved survival by 11% but provided no benefit with regard to local disease control. For low-grade tumors, the risk of death from disease is already low so there would seem to be little role for utilizing chemotherapy for most tumors. One possibility we consider is that the patients in our series receiving systemic therapy may have had erroneous pathologic diagnoses entered into the database. The NCCN guidelines recommend that all patients with STS be evaluated by a multidisciplinary team with experience managing STS. In this setting, experienced surgeons and radiation oncologists can work together to optimize therapy to achieve local control and preserve limb function whenever possible.

Our analysis of patients in the NCDB indicates that radiotherapy does not improve or decrease overall survival in low-grade lesions. This is consistent with the large cohort of study of the SEER database by Koshy et al. examining 2317 patients with “low grade” histology. Of note, tumor grade in this study was categorized into low or high even though sarcomas are typically graded using a three-tier system consisting of low, intermediate, and high grades [25, 26]. A study by Mollabashy et al. looked at a smaller cohort of 108 patients with low grade STS and found that the addition of RT to surgical excision had no effect on either local control or overall survival [27]. The addition of RT had a higher rate of complications as compared to surgery, specifically postoperative lymphedema.

Factors that carry a poor prognostic outcome for soft tissue sarcoma of the extremities include size greater than 5 cm and positive margin status following resection [28]. We examined the effect of these prognostic factors on overall survival in patients with well-differentiated STS-E and found no significant effect on overall survival. Therefore, these results are consistent with tumor grade being the most important prognostic factor for patients with soft tissue sarcoma. There was no overall survival benefit associated with adjuvant radiation therapy when compared to surgery alone in these groups. These findings are in contrast to a small study by Choong et al. They reviewed 132 patients with low-grade STS-E and found that patients with tumors larger than 5 cm and positive margins benefited from the addition of RT with decreased local recurrence and increased metastasis-free survival. Overall survival was not directly addressed [6]. As expected, patients with small lesions (<5 cm) and negative margins showed no benefit in overall survival with the addition of RT. But, in contrast with what has been previously suggested, our data indicates that RT also conferred no survival benefit on patients with large tumors (>5 cm) and resections with positive margins.

In comparing our findings with Choong et al. and Yang et al., important limitation of our study becomes evident: it is not possible to determine rates of local recurrence or metastasis using the NCDB. Local recurrence is thought to be a significant predictor of poor prognosis; particularly in high-grade sarcomas, studies show that patients with local recurrences have higher rates of metastasis and shorter overall survival rates [29, 30]. While RT may not impact overall survival, in combination with limb-sparing surgery moderate doses of RT can eliminate microscopic disease beyond the area of gross resection and reduce rates of local recurrence [20]. The addition of LR and metastasis data to the NCDB would offer two specific utilities. First, it would help to elucidate the role of RT in reducing rates of local recurrence. Second, it would allow investigation of the impact of local recurrence on distant recurrence, disease specific survival, and overall survival in this population of patients with WD tumors.

Another limitation of our study is that it was limited to survival data acquisition prior to 2006. It is possible that trends in RT usage and management have changed in the past 8 years and therefore the outcomes with contemporary RT technology may be different. Indeed, recent reports of IMRT for STS-E suggest increased local control with decreased morbidity [31, 32]. Another limitation of the present study relates to how specimens were obtained for grading. Histology can be evaluated from a core needle biopsy specimen or from the entire tumor specimen following resection. Evaluations from a biopsy could be a source of sampling error, which may affect our results. It is also unclear if tumor grading changed after resection; it is possible that the information entered into the database represents the original biopsy and not the final pathology. We kept this possibility in consideration in our analysis. A small number of patients (125) who received neoadjuvant RT were excluded due to the challenge of grading the tumor following definitive resection as a consequence of radiation effect.

Atypical fatty tumors such as well-differentiated liposarcoma of the extremities remain a unique subset of STS-E with a particularly favorable biological behavior [33, 34]. Though they can be locally aggressive, well-differentiated liposarcomas exhibit lower rates of metastasis [35] and higher rates of overall survival [36, 37]. Radiation therapy is still frequently used in the management of these well-differentiated tumors; this practice remains routine at some institutions [38, 39]. Our study found no significant effect on overall survival with the addition of radiation therapy when compared to surgery alone; this extended to patients with positive margins and patients with tumors greater than 5 cm. Therefore, limb-sparing surgery alone is a reasonable option for well-differentiated liposarcoma of the extremity arising in a location where additional surgical treatment of a local recurrence would not be anticipated to cause significant functional impairment.

Soft tissue sarcomas of the extremities are a diverse and heterogenous group of tumors, which can be challenging to manage. The subset of these tumors that are histologically well-differentiated represents a group of tumors with a more indolent biological behavior. Using a large database, we describe patterns of usage of adjuvant radiation therapy in the United States. In examining this aim, we demonstrate that adjuvant radiation therapy, as expected, does not improve or decrease survival. While radiation therapy will continue to have an important role in reducing the risk of local recurrence for certain well-differentiated STS of the extremities, our results suggest that RT will not impact survival, regardless of tumor size or margin status. Because RT to the extremity can cause short-term toxicity and late effects [40, 41], adjuvant RT should be utilized to treat well-differentiated STS-E when improving local control outweighs these side effects and not to try to improve overall survival [8]. The decision about whether or not to utilize adjuvant RT should be made in the setting of a multidisciplinary discussion with consideration given to each patient's unique situation.

## Figures and Tables

**Table 1:** Baseline characteristics for all patients with well-differentiated tumors.

Variable	Overall (*n* = 2113)	Surgery alone (*n* = 1540)	Adjuvant RT (*n* = 573)	*p* value
*Patient characteristics *				
Age, yrs. (IQR)	55 (43, 68)	55 (43, 69)	54 (43, 67)	0.419
Female	1,035 (48%)	752 (48.1%)	283 (47.8%)	0.957
Race				0.792
White	1,774 (84%)	1,290 (84%)	484 (84%)	
Black	255 (12.1%)	188 (12.2%)	67 (11.6%)	
Other	83 (3.9%)	58 (3.8%)	25 (4.3%)	
Charlson Comorbidity Score				0.777
0	1,265 (88.6%)	908 (88.3%)	357 (89.5%)	
1	140 (9.8%)	103 (10%)	37 (9.3%)	
≥2	22 (1.5%)	17 (1.7%)	5 (1.3%)	
Education above median	1,239 (60.9%)	878 (59.7%)	361 (63.9%)	0.091
Income above median	1,406 (69.1%)	1,005 (68.3%)	401 (71%)	0.269
Distance to cancer center (IQR)	13.4 (5.6, 36.4)	14.7 (5.7, 41.3)	10.9 (5.1, 24.6)	0.611
Treatment facility				0.288
Community Cancer Program	117 (5.5%)	82 (5.4%)	35 (6%)	
Comprehensive Community Cancer Program	827 (39.2%)	585 (38.3%)	242 (41.5%)	
Academic/Research Program	1,165 (55.2%)	859 (56.3%)	306 (52.5%)	
Uninsured	76 (3.7%)	53 (3.5%)	23 (4%)	0.697

*Tumor characteristics *				
Limb location				0.61
Upper limb and shoulder	499 (23.1%)	367 (23.5%)	132 (22.3%)	
Lower limb and hip	1,658 (76.9%)	1,198 (76.5%)	460 (77.7%)	
Tumor size (mm)	90 (40, 170)	84 (35, 170)	97 (50, 159.2)	0.504
Tumor size				<**0.001**
<5 cm	582 (31.3%)	449 (33.9%)	133 (24.7%)	
5–9.9 cm	406 (21.8%)	268 (20.2%)	138 (25.7%)	
10–19.9 cm	527 (28.3%)	344 (26%)	183 (34%)	
>20.0 cm	347 (18.6%)	263 (19.9%)	84 (15.6%)	
Histology				**0.001**
Clear cell sarcoma	4 (0.2%)	4 (0.3%)	0 (0%)	
Epithelioid sarcoma	8 (0.4%)	5 (0.3%)	3 (0.5%)	
Fibrosarcoma	91 (4.2%)	73 (4.7%)	18 (3%)	
Leiomyosarcoma	231 (10.7%)	185 (11.8%)	46 (7.8%)	
Liposarcoma	1,418 (65.7%)	1,014 (64.8%)	404 (68.2%)	
Malignant fibrous histiocytoma	103 (4.8%)	70 (4.5%)	33 (5.6%)	
Mixed mesenchymal sarcoma	3 (0.1%)	3 (0.2%)	0 (0%)	
MPNST	64 (3%)	44 (2.8%)	20 (3.4%)	
Myxosarcoma	19 (0.9%)	9 (0.6%)	10 (1.7%)	
Rhabdomyosarcoma	2 (0.1%)	1 (0.1%)	1 (0.2%)	
Sarcoma NOS	122 (5.7%)	98 (6.3%)	24 (4.1%)	
Small cell sarcoma	2 (0.1%)	0 (0%)	2 (0.3%)	
Spindle cell sarcoma	45 (2.1%)	33 (2.1%)	12 (2%)	
Synovial sarcoma	44 (2%)	25 (1.6%)	19 (3.2%)	
Undifferentiated sarcoma	1 (0%)	1 (0.1%)	0 (0%)	

*Treatment specifics *				
Surgery type				**0.002**
Local excision	723 (33.5%)	525 (33.5%)	198 (33.4%)	
Radication resection	1,365 (63.3%)	976 (62.4%)	389 (65.7%)	
Limb amputation	65 (3%)	60 (3.8%)	5 (0.8%)	
Major amputation	4 (0.2%)	4 (0.3%)	0 (0%)	
Amputation (versus no amputation)	69 (3.2%)	64 (4.1%)	5 (0.8%)	<**0.001**
Days to definitive surgery (IQR)	0 (0, 34)	0 (0, 33)	10 (0, 35.2)	0.598
Neoadjuvant chemo	1 (0.3%)	0 (0%)	1 (1.2%)	0.256
Adjuvant chemo	6 (1.8%)	1 (0.4%)	5 (6%)	**0.005**

*Endpoints and outcomes *				
Surgical margins				**0.001**
Negative	1,534 (78.5%)	1,133 (80.5%)	401 (73.3%)	
Microscopic	234 (12%)	146 (10.4%)	88 (16.1%)	
Macroscopic	186 (9.5%)	128 (9.1%)	58 (10.6%)	
30-day readmission	35 (2.6%)	27 (2.7%)	8 (2.1%)	0.626
Hospital LOS (IQR)	1 (0, 3)	1 (0, 3)	1 (0, 3)	0.427

**Table 2:** Baseline characteristics for patients with well-differentiated liposarcoma only.

Variable	Overall (*n* = 1418)	No RT (*n* = 1014)	Adjuvant only (*n* = 404)	*p* value
*Patient characteristics *				
Age, yrs. (IQR)	57 (45, 70)	57 (46, 70)	54 (44, 68)	**0.006**
Female	676 (47.7%)	484 (47.7%)	192 (47.5%)	0.991
Race				0.875
White	1,171 (84.2%)	837 (84%)	334 (84.6%)	
Black	160 (11.5%)	117 (11.7%)	43 (10.9%)	
Other	60 (4.3%)	42 (4.2%)	18 (4.6%)	
Charlson Comorbidity Score				0.791
0—none	836 (88.2%)	600 (88%)	236 (88.7%)	
1—one point	97 (10.2%)	72 (10.6%)	25 (9.4%)	
2—two or more points	15 (1.6%)	10 (1.5%)	5 (1.9%)	
Education above median	820 (61.2%)	576 (60.4%)	244 (63.2%)	0.367
Income above median	922 (68.8%)	647 (67.8%)	275 (71.2%)	0.246
Distance to cancer center (IQR)	14.2 (5.8, 39.5)	15.5 (6.2, 44.5)	11 (5.3, 26.8)	0.867
Treatment facility				**0.021**
Community Cancer Program	74 (5.3%)	47 (4.8%)	27 (6.8%)	
Comprehensive Community Cancer Program	534 (38.5%)	364 (36.8%)	170 (42.6%)	
Academic/Research Program	780 (56.2%)	578 (58.4%)	202 (50.6%)	
Uninsured	43 (3.1%)	30 (3.1%)	13 (3.3%)	0.956

*Tumor characteristics *				
Limb location				0.856
Upper limb and shoulder	212 (15%)	150 (14.8%)	62 (15.3%)	
Lower limb and hip	1,206 (85%)	864 (85.2%)	342 (84.7%)	
Tumor size (mm)	130 (70, 200)	133 (70, 200)	121 (71.5, 185)	**0.009**
Tumor size				**0.002**
5 cm	197 (15.7%)	149 (16.9%)	48 (12.8%)	
5–9.9 cm	262 (20.9%)	173 (19.7%)	89 (23.7%)	
10.0–19.9 cm	457 (36.4%)	300 (34.1%)	157 (41.9%)	
>20.0 cm	339 (27%)	258 (29.3%)	81 (21.6%)	
Histology				0.999
Liposarcoma	1,418 (100%)	1,014 (100%)	404 (100%)	

*Treatment specifics *				
Surgery type				0.439
Local excision	461 (32.5%)	337 (33.2%)	124 (30.7%)	
Radical resection	938 (66.1%)	661 (65.2%)	277 (68.6%)	
Limb amputation	18 (1.3%)	15 (1.5%)	3 (0.7%)	0.31
Major amputation	1 (0.1%)	1 (0.1%)	0 (0%)	
Days to definitive surgery (IQR)	0 (0, 27)	0 (0, 24)	9 (0, 34.5)	0.393

*Selection of adjuvant RT by surgical margin *				
Surgical margins				**0.014**
Negative	946 (74.9%)	684 (76.7%)	262 (70.6%)	
Positive margin, microscopic	182 (14.4%)	112 (12.6%)	70 (18.9%)	
Positive margin, macroscopic	135 (10.7%)	96 (10.8%)	39 (10.5%)	
30-day readmission	25 (2.7%)	21 (3.2%)	4 (1.6%)	0.257
Hospital LOS (IQR)	1 (0, 3)	1 (0, 3)	1 (0, 3)	0.452

**Table 3:** Five year propensity matched survival.

Grouping	Number of patients	Surgery only	Adjuvant RT	*p* value
*Well-differentiated tumors* when stratified by adjuvant RT versus surgery alone	1146	92.2% (89.9–94.6%)	89.5% (86.9–92.2%)	0.984

*Well-differentiated tumors* when stratified by adjuvant RT versus surgery on the basis of negative margins	772	94.5% (92–97%)	91.6% (88.7–94.6%)	0.85

*Well-differentiated tumors* when stratified by adjuvant RT versus surgery on the basis of positive margins	286	92.4% (88–97.1%)	84.4% (78.5–90.8%)	0.096

*Well-differentiated liposarcoma* when stratified by adjuvant RT versus surgery alone	808	91.7% (88.8–94.7%)	90% (87–93.1%)	0.84

*Well-differentiated liposarcoma* when stratified by adjuvant RT versus surgery on the basis of negative margins	524	93.7% (90.6–97%)	91.3% (87.7–95%)	0.891

*Well-differentiated liposarcoma* when stratified by adjuvant RT versus surgery on the basis of positive margins	218	90.8% (85.3–96.7%)	87.5% (81.4–94.1%)	0.735
